# Problematic Use of Smartphones and Sleep Quality Among Healthcare Workers in Qassim, Saudi Arabia

**DOI:** 10.7759/cureus.63610

**Published:** 2024-07-01

**Authors:** Muath M Aldukhayel, Unaib Rabbani

**Affiliations:** 1 Family Medicine Academy, Qassim Health Cluster, Buraydah, SAU

**Keywords:** sleep, smartphones, saudi arabia, mental health, healthcare workers, addiction

## Abstract

Background and objectives

Problematic use of smartphones among healthcare workers can affect their performance, patient care, safety, care outcomes, and patient satisfaction. The aim of this study was to determine the prevalence of problematic use of smartphones and the relationship between the problematic use of smartphones and sleep quality among healthcare workers in Qassim, Saudi Arabia.

Methods

We enrolled 393 healthcare workers conveniently selected online for this cross-sectional survey. We assessed the problematic use of smartphones using the short version of the Smartphone Addiction Scale. For sleep quality, we used the Pittsburgh Sleep Quality Index (PSQI). Linear regression was used to assess the association of problematic use of smartphones with sleep quality. IBM SPSS Statistics, version 23.0 (IBM Corp., Armonk, NY) was used for analyses.

Results

The prevalence of smartphone addiction (SMA) was 59.0%, and 30.5% were at high risk for addiction. The mean PSQI score was 11.56 ± 2.1 out of 21. It was found that female gender was associated with poor sleep quality (adjusted B = 0.45, p-value = 0.049). On the other hand, SMA was also significantly associated with poor sleep quality (adjusted B = 0.90, p-value = 0.016).

Conclusion

There is a high prevalence of problematic use of smartphones among healthcare workers, which is associated with poor sleep quality. Given the significant occurrence of problematic smartphone use among healthcare professionals and its detrimental effects on sleep quality, it is crucial for public health initiatives to devise and execute suitable preventive measures, such as smartphone use policies at work and education of workers.

## Introduction

The widespread use of personal mobile phones has become essential in our daily lives. In the medical field, the increasing use of medical apps enables healthcare professionals to communicate more efficiently and access important information quickly, ultimately enhancing patient care. However, using smartphones during work can lead to distractions and errors in healthcare procedures.

Often, doctors are “digital omnivores,” using multiple devices routinely in a professional setting, and the number of healthcare professionals who use even three digital platforms at the same time (smartphone, tablet, and laptop/desktop) has been projected to increase [1]. The percentage of healthcare professionals using smartphones has risen from 66% to 90% in 2012 [2].

The problematic use of smartphones has been linked to various health outcomes. According to a systematic review, there was a consistent correlation between problematic smartphone use and stress, anxiety, depression, and self-esteem [3]. Anxiety was significantly associated with problematic smartphone use and sleep disturbance among medical students during the COVID-19 pandemic. Problematic smartphone use not only directly affected anxiety but also exerted a significant indirect effect on anxiety via sleep disturbance, according to a study on Chinese medical students [4]

According to a cross-sectional study among medical students, addiction to smartphones was associated with variant sleep disorders, such as insomnia, interrupted sleep, and early morning wakeup [5]. This study found a strong and significant relationship between sleep problems and smartphone addiction (SMA). The most common sleep complication among the high-risk group was feeling sleepy during work. The prevalence of insomnia in our study was 28.9% [6].

Evidence also suggests that poor outcomes are associated with problematic smartphone use among medical and nursing students. A local study raised the possibility that problematic smartphone use resulted in a negative impact on academic achievement, eating habits, mood, energy level, and sleep [7]. Another study found that SMA and problematic use of smartphones were linked to an increased risk of poor quality of sleep and reduction in academic performance among medical students at King Abdulaziz University (KAU), Jeddah, Saudi Arabia [8]. A systematic evaluation among nursing students reveals that smartphone use was greatly expanded by nursing students during clinical practice. They mentioned that they were using the gadget or observed other pupils being preoccupied with their smartphones. Lower sleep quality, lower self-esteem, more social discomfort, lower perceived social support, or poorer interpersonal communication skills were some factors associated with excessive usage among nursing students [9].

The problematic use of smartphones is associated with various mental and sleep disorders, which affect performance. Such problematic use among healthcare workers can also affect their performance, patient care, safety, care outcomes, and patient satisfaction. This study aimed to measure the current level of use of smartphones among healthcare workers and its relationship with sleep quality in Qassim, Saudi Arabia.

## Materials and methods

Study design and population

Based on a cross-sectional design, this study was conducted among healthcare workers in Qassim. The healthcare workers included physicians, dentists, nurses, midwives, pharmacists, lab and radiology technicians, dietitians, and administrative staff. 

Sample size

The sample size was calculated using the OpenEpi sample size calculator [10]. An expected prevalence of 64% for problematic use of smartphones was considered based on a study conducted among the adult population in the Qassim region [11]. At a 95% confidence level with 5% absolute precision, the required sample size was 354. To adjust for missing information, the sample was inflated by 10%; therefore, the final sample required was 390 healthcare workers.

Sampling procedure

Participants were selected conveniently online. The link to the questionnaire was shared among the staff of all healthcare facilities (public and private) through social media portals (WhatsApp, Twitter, and Instagram). Contact was also made to the administrations of healthcare facilities, requesting them to share the questionnaire among the staff.

Inclusion and exclusion criteria

We included all current healthcare workers of either gender in the Qassim region. Interns, temporary, and ad-hoc healthcare workers were excluded from the study.

Data collection tool

Data were collected using an anonymous structured questionnaire online in English and Arabic. The questionnaire was developed on Google Forms. We checked the questionnaire for wording and understanding. This was followed by a review of the questionnaire by public health experts and then a pilot study on a few selected healthcare workers of different cadres. Following this, the actual data collection was commenced. The questionnaire included sections on socio-demographic and professional profiles, Pittsburgh Sleep Quality Index (PSQI), and Smartphone Addiction Scale Short Version, Quality Score.

*The Pittsburgh Sleep Quality Index *[12]

It consists of 17 self-rated questions that are grouped into seven rated components: (1) sleep quality, (2) sleep latency, (3) habitual sleep efficiency, (4) sleep duration, (5) use of sleeping medication, (6) sleep disturbances, and (7) daytime dysfunction. To calculate the score of the questionnaire, each question is assigned a score from 0 to 3. The item scores are used in calculating the seven component scores, which are then added to produce a total score that can range from 0 to 21, where a higher score indicates poor sleep quality. If the total score obtained is 5 or greater, it suggests poor sleep quality. Moreover, if it was less than 5, then this is considered to be good sleep quality. We also used the Arabic version of PSQI, which has been validated previously [13]. Due to contextual factors and feasibility issues, we did not include five questions rated by the bed partner or roommate. However, these questions are not included in the calculations of any of the seven components. Therefore, the validity of the questionnaire is not affected.

*‏Smartphone Addiction Scale Short Version, Quality Score *[14]

This scale assesses daily-life disturbance, withdrawal, overuse, and tolerance of using a smartphone. The Arabic version is also used, which has been validated previously [15]. This scale consists of 10 items measured on a six-point Likert scale as the following: (1) “strongly disagree,” (2) “disagree,” (3) “slightly disagree,” (4) “slightly agree,” (5) “agree,” and (6) “strongly agree.” The maximum possible score is 60, where higher scores indicate an addiction. The cut-offs that define the high risk of addiction and addiction for males and females are different. Males are considered addicted if scores are higher than 31. A high risk of addiction is present, with scores between 22 and 31. Females are addicted if scores are higher than 33 and at high risk with scores between 22 and 33.

The first page of the online questionnaire contained information about the research process and principal investigator, along with contact details and an informed consent agreement. The survey would proceed only if the participant provided consent.

Data analysis and management

Data were organized, coded, and analyzed using IBM SPSS Statistics, version 23.0 (IBM Corp., Armonk, NY). Descriptive statistics were performed to calculate percentages and frequencies for categorical variables, while continuous variables were presented as mean and standard deviation. The dependent variable in the current study was sleep quality, while problematic smartphone use and other socio-demographic variables were independent variables. Inferential statistics in the form of chi-square, ANOVA, and t-test were performed to compare and test for association between different study variables. Univariate and multivariable linear regression was used to assess the association of sleep quality score with SMA and other variables. K-1 dummy variables were created for categorical variables, where K indicates the number of categories in the variable. A p-value of <0.05 was considered statistically significant.

Ethical approval

Ethical approval was obtained from the Regional Research Ethics Committee, Qassim province No. 607/44/12551 on March 15, 2023. Informed consent was taken from each of the participants. Confidentiality was maintained, and information was not shared to any agency.

## Results

The study enrolled 393 participants. The mean (±SD) of the age was 36.6 ± 8.4. A little more than 50% of the participants were males, and nearly three-quarters were Saudi. The majority of the participants were from Buraydah (60.1%) and Unayzah (23.4%) and had bachelor’s degrees (57.8%). Moreover, 56.2% were working in the hospital, and 41.4% were in outpatient departments.

There was a weak but significant correlation of age with PSQI score (Pearson correlation coefficient, 0.129), with a p-value of 0.011. The Saudi nationals had significantly higher PSQI scores (11.7 ± 2.1) as compared to non-Saudi healthcare workers (11.3 ± 2.1), with a p-value of 0.044. The pharmacist had a significantly higher PSQI score (12.0 ±1.8) than others, with a p-value of 0.044. We did not find a significant difference in PSQI scores with respect to the education level, city, and place of work (Table 1).

**Table 1:** Socio-demographic and professional profile of participants and Pittsburgh Sleep Quality Index (PSQI) score

Variable	% (n)	PSQI score Mean (SD)	p-value
Age			
Pearson correlation	36.6 (8.4)	0.129	0.011
Gender (n = 392)			
Male	56.4 (221)	11.4 (2.2)	0.054
Female	43.6 (171)	11.8 (2.0)	
Nationality			
Saudi	61.8 (243)	11.7 (2.1)	0.041
Non-Saudi	38.2 (150)	11.3 (2.1)	
Job type			
Doctor	40.6 (159)	11.2 (2.2)	0.044
Trainee/fellow	12.0 (47)	11.8 (2.3)	
Nurse	23.0 (90)	11.9 (1.9)	
Pharmacist	7.4 (29)	12.0 (1.8)	
others	10.2 (40)	11.8 (2.1)	
Administrator/manager	6.9 (27)	11.4 (2.1)	
Education			
Bachelor (MD, MBBS, or similar)	57.8 (227)	11.7 (2.1)	0.452
Diploma	9.4 (37)	11.6 (2.0)	
Master	15.0 (59)	11.2 (2.5)	
PhD or board	17.8 (70)	11.5 (1.9)	
City			
Buraydah	60.1 (236)	11.4 (2.1)	0.147
Unayzah	23.4 (92)	11.7 (1.8)	
Alrass	11.7 (46)	12.1 (2.4)	
Others	5 (19)	11.3 (2.7)	
Place of work			
Hospital	56.2 (221)	11.7 (2.1)	0.352
Primary health care center	36.9 (145)	11.4 (2.1)	
Administration/cluster/directorate	6.9 (27)	11.2 (2.3)	
Department in hospital (n = 220)			
Inpatient	29.5 (65)	11.7 (2.1)	0.644
Outpatient	41.4 (91)	11.6 (2.0)	
Emergency	25.5 (56)	12.0 (2.1)	
Administration	3.6 (8)	11.1 (3.5)	

**Figure 1:**
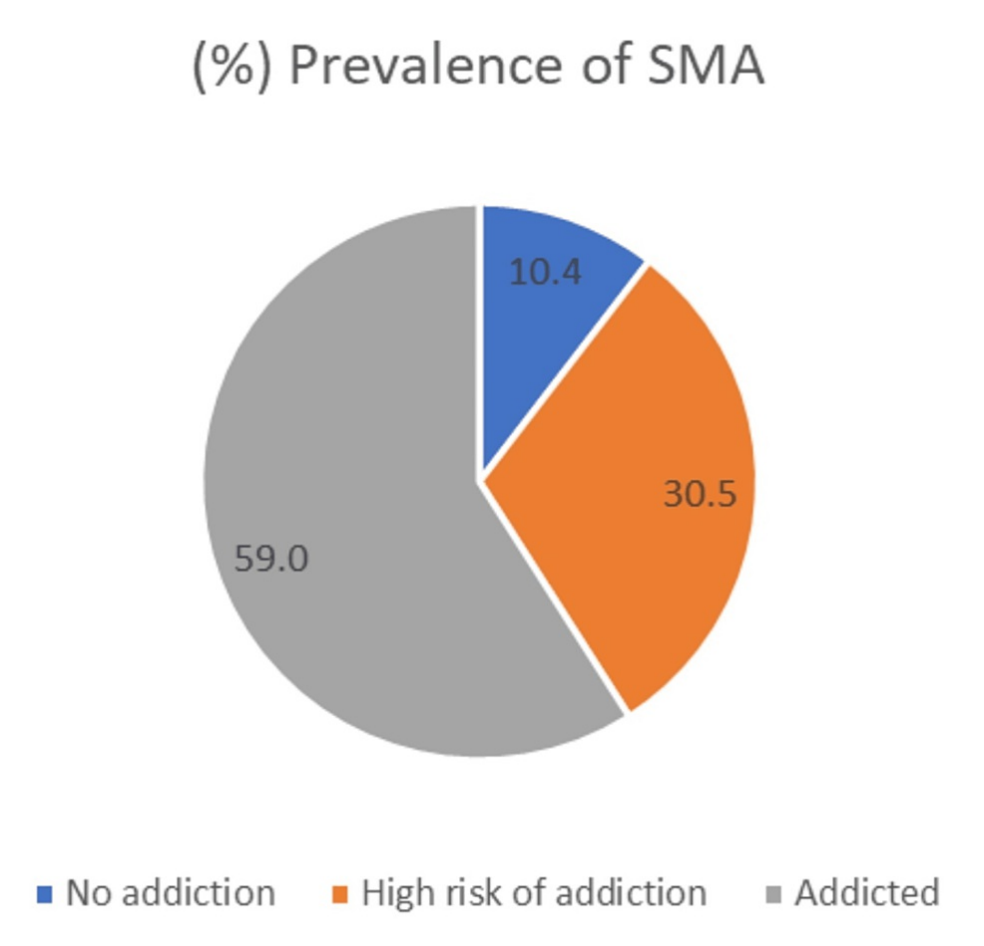
Prevalence of smart phone addiction (SMA) among healthcare workers in Qassim, Saudi Arabia (n = 393)

The prevalence of SMA was 59.0%, and the high risk for addiction was 30.5%, while no addiction was found at 10.4% (Figure 1). The mean PSQI score was 11.56 ± 2.1.

Table 2 shows the association of SMA and other characteristics of the participants with sleep quality. In the univariate analyses, there was a negative association between age and PSQI score (B = −0.032, p-value = 0.011). Being non-Saudi was associated with a lower PSQI score than Saudi (B = −0.448, p-value = 0.041). There is a positive association between nurses and PSQI scores as compared to doctors (B = 0.758, p-value = 0.006). Also, the same association was found with pharmacists (B = 0.867, p-value = 0.039). No association was found with the education level. There was a positive association between Alrass city and PSQI score as compared to Buraydah (B = 0.730, p-value = 0.032). Place of work showed no significant association.

In the multivariable analysis, there was a positive association between female gender and PSQI score (B = 0.478, p-value = 0.049). We did not find a significant difference in PSQI scores with respect to age, nationality, job type, education, city, and place of work. There was a significant association between SMA and PSQI score (B = 0.983, p-value = 0.006) in univariate analyses, and this association remained significant in the multivariable model after adjusting for the confounding effect of other variables (B = 0.899, p-value = 0.016) (Table 2).

**Table 2:** Association of Pittsburgh Sleep Quality Index (PSQI) score with smartphone addiction and personal characteristics among healthcare workers

Variables	Univariate		Multivariable	
	Crude B (95% CI)	p-value	Adjusted B (95% CI)	p-value
Age	-0.032 (-0.056 to -0.008)	0.011	-0.008 (-0.039 to 0.022)	0.584
Gender				
Male	1		1	
Female	0.414 (-0.008 to 0.837)	0.054	0.478 (0.002 to 0.953)	0.049
Nationality				
Saudi	1		1	
Non-Saudi	-0.448 (-0.879 to -0.018)	0.041	-0.290 (-0.838 to 0.258)	0.229
Job type				
Doctor	1		1	
Trainee/fellow	0.672 (-0.013 to 1.356)	0.055	0.174 (-0.584 to 0.932)	0.652
Nurse	0.758 (0.214 to 1.303)	0.006	0.519 (-0.109 to 1.146)	0.105
Pharmacist	0.876 (0.044 to 1.709)	0.039	0.762 (-0.110 to 1.635)	0.087
others	0.667 (-0.063 to 1.396)	0.073	0.339 (-0.469 to 1.148)	0.41
Administrator/manager	0.212 (-0.646 to 1.070)	0.627	0.007 (-0.867 to 0.881)	0.988
Education				
Bachelor (MD, MBBS, or similar)	1		1	
Diploma	-0.117 (-0.854 to 0.620)	0.755	-0.257 (-0.993 to 0.479)	0.493
Master	-0.502 (-1.110 to 0.106)	0.106	-0.473 (-1.122 to 0.176)	0.153
PhD or board	-0.128 (-0.697 to 0.441)	0.658	0.292 (-0.346 to 0.930)	0.369
City				
Buraydah	1		1	
Unayzah	0.295 (-0.215 to 0.805)	0.256	0.226 (-0.282 to 0.734)	0.382
Alrass	0.730 (0.062 to 1.398)	0.032	0.522 (-0.165 to 1.209)	0.136
Others	-0.109 (-1.097 to 0.879)	0.829	0.182 (-0.780 to 1.144)	0.71
Place of work				
Hospital	1			
Primary health care center	-0.245 (-0.691 to 0.200)	0.279		
Administration/cluster/directorate	-0.501 (-1.348 to 0.346)	0.245		
Smartphone addiction				
No addiction	1		1	
High risk of addiction	0.137 (-0.614 to 0.888)	0.72	0.111 (-0.642 to 0.864)	0.772
Addicted	0.983 (0.278 to 1.689)	0.006	0.899 (0.166 to 1.632)	0.016

## Discussion

The primary aim of this cross-sectional study was to assess the prevalence of SMA and its association with sleep quality among healthcare workers in Qassim. In contrast to the predominant focus of previous research on smartphone use and sleep quality within the general population and students, our investigation concentrates on healthcare professionals.

Our study, reporting an SMA prevalence of 59%, reflects concerns raised in similar local studies among adults in Qassim, dental students in Saudi Arabia, and medical students at KAU, where participants exhibited addictive behaviors with prevalences of 64% [11], 71.9% [16], and 73.4% [9], respectively. On the contrary, other local studies have shown a lower percentage of SMA. For example, addictive behaviors were found in 48% of a group of King Saud University students [17], 36.5% among sixth-year medical students at the faculty of medicine of KAU [18], and 19.1% among college and university students in Saudi Arabia in all provinces [6]. A study conducted in four countries in the Middle East showed different prevalence of problematic smartphone use: in Jordan, 59.8%; in KSA, 27.2%; in Sudan, 17.3%; and in Yemen, 8.6% [19]. In other countries, studies have reported different prevalence: 38.9% in the United Kingdom [20], 38.5% in China [21], almost 30% in Malaysia [22], and 12.5% in Spain [23]. The differences in the prevalence of SMA in Saudi Arabia can be attributed to various factors, like study methodologies, sample size, and population demographics. Furthermore, socioeconomic factors can impact the rates, as those with lower socioeconomic status may have limited access to smartphones. Additionally, differences in awareness and recognition of SMA as a problem can also contribute to varying prevalence rates. The timeframe of the studies can affect the result as the prevalence may vary over time as technology advances and societal norms shift. Moreover, the introduction of new features, applications, and social media platforms can influence SMA rates. It is strongly recommended that health authorities should conduct educational and awareness programs about the risk of excessive smartphone use and ways to control excessive use. Workplace smartphone use policies are also required to reduce the unnecessary use of smartphones in the workplace. Implementing these strategies can help address SMA among healthcare workers and improve their well-being and performance.

An observation emerged: there is a higher addiction and poorer sleep quality among females compared to males (p-value = 0.049). This outcome aligns with prior research conducted by Ibrahim et al. (p-value < 0.001) [8]. The observed pattern could be attributed to the commonly observed inclination of females toward engaging in conversations and interpersonal interactions, as opposed to males [9]. In contrast, studies conducted by Aljomaa et al. [17] and Alhazmi et al. [18] unveiled a pattern where males exhibited a higher frequency of smartphone usage, while another study conducted by Al-Mohaimeed et al. found no statistically significant association between gender and problematic smartphone use. Age and marital status could potentially contribute to the variance between the studies, as older and married women may have had additional responsibilities that kept them occupied compared to the students [11].

We also observed that younger age strongly predicts high PSQI scores associated with higher SMA, which was also reported by a local study among college and university students in Saudi Arabia [6], and a Chinese study among adults predicted the association between age and SMA [21]. This may be due to the widespread smartphone use among younger individuals, involving diverse activities like social media interactions, entertainment, and communication.

This study significantly contributes to the growing body of evidence highlighting the adverse effects of SMA on sleep quality. Previous research has established a direct link between SMA and sleep disturbances, as reported by Sohn et al. among young adults in the UK [20] and Ibrahim et al. in Saudi Arabia [9]. Furthermore, the strong association between SMA and mental health issues, including depression (p-value < 0.001) and anxiety (p-value = 0.001), underscores the multifaceted impact of excessive smartphone usage on overall well-being [24]. This is important for healthcare providers and planners because poor sleep can negatively affect the performance of workers and thus lead to poor patient outcomes.

Our study underscores the importance of addressing smartphones’ impact on sleep quality among healthcare workers. We used validated measurement tools to assess the main variables, i.e., SMA and sleep quality. A diverse group of healthcare workers was included to generate a more representative sample. However, there are some limitations. First, the cross-sectional design does not allow for conclusions about the direction of causality between the SMA and sleep quality. Second, the reliance on self-reported data introduces the potential for social desirability bias. However, we collected data in a completely anonymized way; therefore, we assume that respondents would have given accurate responses. Third, variables such as psychological distress and job stress were not controlled for, which may be correlated with sleep problems and problematic smartphone use.

## Conclusions

In our study, the overall occurrence of problematic smartphone use was high among participants (59.0%). There was a significant association between SMA and a decrease in sleep quality. Since the problematic use of smartphones is highly prevalent among healthcare workers and has an impact on sleep quality, there is a need for clear guidelines on smartphone usage during work, educational programs about healthy smartphone habits, and promoting work-life balance. Additional research should prioritize exploring the correlation between the quality of life related to health and the problematic usage of smartphones.
